# Study on the combined application of selected virtual monoenergetic imaging, virtual non-contrast imaging, and adaptive statistical iterative reconstruction-V in head and neck CT angiography using 256-slice wide-body CT

**DOI:** 10.3389/fmed.2026.1872871

**Published:** 2026-08-24

**Authors:** Wen Chao Li, Pei Heng Li, Qiang Li, Ran Song, Ming Mei Xue, Pei Ru Li, Chang Hua Liang

**Affiliations:** Department of Radiology, The First Affiliated Hospital of Xinxiang Medical University, Weihui, Xinxiang, China

**Keywords:** CT angiography, iodinated contrast medium, iterative reconstruction, radiation dose, virtual monoenergetic imaging, virtual non-contrast imaging

## Abstract

**Objective:**

To evaluate the value of combining spectral CT-derived virtual monoenergetic imaging, adaptive statistical iterative reconstruction-V (ASIR-V), and virtual non-contrast imaging in head and neck CT angiography (CTA) for patients with cerebrovascular disease (CVD).

**Methods:**

150 patients undergoing head and neck CTA for suspected or known CVD were randomly assigned 1:1:1 to the conventional-dose, spectral low-dose, and spectral ultra-low-dose groups. The conventional-dose group underwent a true non-contrast scan followed by 100-kVp polychromatic CTA with filtered back projection (FBP) reconstruction and received 0.90 mL/kg contrast at 5.0 mL/s, whereas the two spectral groups underwent a single GSI CTA acquisition generating virtual non-contrast images. Reported conventional-group DLP and ED included both acquisitions. The spectral low-dose group used 65-keV, 50% ASIR-V reconstruction and received 0.55 mL/kg at 4.5 mL/s, whereas the ultra-low-dose group used 60-keV, 70% ASIR-V reconstruction and received 0.50 mL/kg at 4.0 mL/s. Vascular CT attenuation, signal-to-noise ratio (SNR), contrast-to-noise ratio (CNR), subjective image quality, dose-length product (DLP), effective dose (ED), total contrast agent volume, and total iodine load were compared among groups.

**Results:**

CT values of the aortic arch, common carotid artery, internal carotid artery, and M1 segment, and cervical and cerebral vascular SNR and CNR, were lower in both spectral groups than in the conventional-dose group (*p* < 0.05). No significant differences were observed between the spectral low-dose and spectral ultra-low-dose groups (*p* > 0.05). Image-quality scores were higher in both spectral groups than in the conventional-dose group, despite their lower vascular attenuation, SNR, and CNR (*p* < 0.05). The DLP, ED, actual total contrast agent volume, and total iodine load were significantly lower in both spectral groups than in the conventional-dose group (*p* < 0.05). The spectral ultra-low-dose group showed a statistically significant further reduction in actual total contrast agent volume and the derived total iodine load.

**Conclusion:**

The integrated spectral CT protocols incorporating selected virtual monoenergetic imaging, ASIR-V reconstruction, and virtual non-contrast imaging were associated with lower radiation exposure and contrast agent use while maintaining diagnostically acceptable image quality, supporting the preliminary feasibility of the evaluated dose-reduction protocols in head and neck CTA.

## 1 Introduction

Cerebrovascular disease remains a major cause of death and long-term disability worldwide, including in China. Cerebrovascular disease encompasses a heterogeneous group of ischemic and hemorrhagic disorders affecting the cerebral circulation. Its occurrence is closely associated with factors such as age, dietary habits, and living environment, and it exhibits considerable etiological and genetic diversity. The progression of cerebrovascular disease is often rapid, and because acute cerebral ischemia or hemorrhage may cause irreversible neurological injury, patients are at high risk of permanent disability or death once the disease occurs. Early, accurate diagnosis and prompt treatment are therefore essential to reducing the incidence, disability, and mortality associated with cerebrovascular disease (CVD) (1, 2).

In clinical practice, the primary diagnostic modalities for evaluating vascular lesions of the head and neck include transcranial Doppler ultrasonography (TCD), carotid ultrasound, computed tomographic angiography (CTA), digital subtraction angiography (DSA), and magnetic resonance angiography (MRA). However, MRA is contraindicated in patients with metallic implants such as prostheses or aneurysm clips due to the underlying imaging mechanism. Moreover, the relatively long acquisition and immobilization times make it unsuitable for patients with acute cerebrovascular events, postoperative follow-up needs, or severe conditions that limit cooperation. Carotid and transcranial ultrasound are non-invasive and radiation-free, but their diagnostic accuracy depends heavily on the operator’s skill and experience. In cases of severe stenosis or vessel occlusion, reliable assessment can only be achieved by highly experienced specialists. Furthermore, ultrasound provides limited accuracy in evaluating and localizing lesions in the posterior cerebral circulation. DSA offers a distinct advantage in that it enables immediate therapeutic intervention. Nonetheless, it has notable drawbacks, including its invasive nature, inability to visualize distal vessels beyond occlusion, high radiation exposure, and substantial procedural costs, which collectively limit its use as a routine diagnostic tool. In recent years, CTA has become widely used in vascular imaging. Head and neck CTA not only provides detailed visualization of vascular anatomy but also allows for quantification of arterial blood flow, facilitating accurate diagnosis of cerebrovascular disease (CVD). Its high spatial and contrast resolution enables clear, multidimensional visualization of vessel wall pathology, thrombosis, calcification, and surrounding structures, offering substantial clinical value (1). However, conventional CTA is associated with high radiation exposure and requires large volumes of iodinated contrast, which may pose health risks to patients. With advances in medical science and growing public awareness, increasing attention has been directed toward the potential risks of ionizing radiation from CT imaging. In the late 1970s, the International Commission on Radiological Protection (ICRP) proposed three fundamental principles of radiation protection: justification of practice, optimization of protection, and dose limitation (3). A European study published in 2016 reported a clear linear relationship between radiation exposure from X-ray–based diagnostic and therapeutic procedures and cancer incidence (4). Because head and neck CTA covers a broad anatomical range, sensitive organs such as the lens, thyroid, and cervical glands are directly exposed to ionizing radiation. Evidence indicates that higher radiation doses from CT examinations are associated with an increased risk of cataract formation (5). Therefore, achieving diagnostic-quality images while minimizing both radiation and prescribed weight-based contrast dose has become a key focus in contemporary CT imaging research.

Gemstone Spectral Imaging (GSI) uses rapid switching between high and low tube voltages to acquire dual-energy projection data, enabling the subsequent reconstruction of 101 virtual monoenergetic image sets ranging from 40 to 140 keV. The radiation output remains determined by the acquisition parameters (6). At lower energy levels (approximately 33.2 keV), iodine exhibits a strong K-edge absorption effect, enhancing vascular contrast. This feature enables reductions in both contrast agent volume and contrast injection rate without compromising image visibility. However, at lower virtual monoenergetic energies, statistical fluctuations propagated through spectral material decomposition and image reconstruction may be amplified, resulting in increased image noise. Adaptive statistical iterative reconstruction-V (ASIR-V) can reduce image noise and help preserve diagnostically acceptable image quality in lower-dose acquisitions (7). Virtual non-contrast (VNC) imaging applies iodine-based material decomposition to remove the contrast signal from enhanced CT images, generating virtual unenhanced images. This reduces the need for additional scans and consequently lowers radiation exposure (8, 9). To date, few studies have explored the combined use of selected virtual monoenergetic imaging, virtual non-contrast reconstruction, and ASIR-V technology in spectral CT to simultaneously reduce contrast volume and contrast injection rate. This study aims to evaluate the feasibility of this combined approach in head and neck CTA, seeking to minimize radiation dose, contrast use, and iodine delivery while maintaining diagnostic image quality, thereby providing a reference for clinical optimization of CTA protocols.

## 2 Materials and methods

### 2.1 Clinical data

A total of 150 patients with CVD who underwent head and neck CTA at the First Affiliated Hospital of Xinxiang Medical University between June 15, 2022, and October 15, 2025, were prospectively enrolled. Inclusion criteria were: (1) age 18–90 years, any sex; (2) body mass index (BMI) between 18 and 31 kg/m^2^; (3) clinically suspected or imaging-confirmed CVD, including ischemic stroke, arterial spasm, or atherosclerosis of head and neck vessels, meeting CTA indications; and (4) provision of written informed consent. Exclusion criteria included: (1) history of allergy to iodinated contrast agents; (2) myocardial infarction or severe cardiac dysfunction; (3) hepatic or renal insufficiency; (4) pregnancy or lactation; and (5) impaired consciousness preventing cooperation during the procedure.

#### 2.1.1 Sample size estimation

*A priori* power analysis was performed for the prespecified primary outcome, cervical-vessel contrast-to-noise ratio (CNR), using G*Power version 3.1.9.7 and a fixed-effects one-way ANOVA omnibus test (10). Pilot data showed expected group means of 52.0, 47.0, and 48.0, with a pooled standard deviation of 6.49, corresponding to a Cohen’s f of 0.333. With three groups, *α* = 0.05, and 80% power, at least 90 participants were required, or 30 per group. The target sample size was increased to 150 participants to improve estimate precision and account for potentially non-evaluable examinations. A total of 150 participants were randomly assigned in a 1:1:1 ratio using a computer-generated randomization table to the conventional-dose, spectral low-dose, and spectral ultra-low-dose groups, with 50 participants in each group. Allocation concealment was ensured by a research nurse. This study was conducted in accordance with the Declaration of Helsinki and approved by the Ethics Committee of the First Affiliated Hospital of Xinxiang Medical University (Approval No.: 2020116). Written informed consent was obtained from all participants prior to enrollment, after they were provided with detailed information about the study objectives, procedures, potential risks, and benefits.

### 2.2 CTA examination

CT scans were performed using a GE Revolution CT system (GE Healthcare, USA). Unless otherwise specified, the scanner, patient positioning, anatomical coverage, acquisition parameters, contrast medium, contrast injector, and bolus-tracking procedure described below were applied to both the 20-patient pilot cohort and the 150-patient formal study cohort. All patients were positioned supine, and the scanning range extended from 2–3 cm below the aortic arch to the cranial vertex. Scanning parameters included automatic tube current modulation with a fixed noise index of 10, detector configuration of 256 × 0.625 mm, pitch 1.375:1, gantry rotation time 0.5 s/rev, field of view 25 × 25 cm, and slice thickness 0.625 mm. Iopromide injection (Ultravist 370, 370 mg iodine/mL; Bayer AG, Berlin, Germany) was administered through an antecubital vein using an ImaStar dual-head high-pressure CT contrast injector (Shenzhen Antmed Co., Ltd., Shenzhen, China). The trigger point was set at the common carotid artery at the C4 vertebral level with a threshold of 100 HU. Automatic bolus tracking initiated the scan 3 s after the attenuation threshold of 100 HU was reached. Patients were instructed to avoid swallowing during image acquisition. No prospective or retrospective gating was applied, and no dedicated motion-tracking, image-registration, or retrospective motion-correction algorithm was used. Patient-motion control relied on standard head positioning and instructions to avoid swallowing during the scan. Conventional-dose group: Conventional polychromatic CTA data were acquired at 100 kVp and reconstructed using filtered back projection (FBP). Contrast administration was individualized according to each participant’s measured pre-scan body weight. The prescribed weight-based contrast doses were 0.90, 0.55, and 0.50 mL/kg for the conventional-dose, spectral low-dose, and spectral ultra-low-dose groups, respectively. The prescribed weight-based contrast dose, expressed in mL/kg, represents the volume of contrast medium administered per kilogram of body weight. For each participant, the actual total contrast agent volume, expressed in mL, was calculated as follows: actual total contrast agent volume (mL) = group-specific prescribed weight-based contrast dose (mL/kg) × measured pre-scan body weight (kg).

#### 2.2.1 Pilot parameter-optimization study

Before formal participant enrollment, a pilot parameter-optimization study was conducted in 20 patients who were not included in the final study cohort. The pilot examinations used the same scanner, patient positioning, anatomical coverage, acquisition parameters, contrast medium, contrast injector, and bolus-tracking procedure described above. All spectral pilot examinations were acquired using rapid 80/140-kVp switching GSI mode. Of the 20 pilot patients, 10 received a prescribed weight-based contrast dose of 0.55 mL/kg at an injection rate of 4.5 mL/s, whereas the remaining 10 received 0.50 mL/kg at an injection rate of 4.0 mL/s. For each spectral acquisition, virtual monoenergetic images were reconstructed at 50, 55, 60, 65, and 70 keV, with ASIR-V blending strengths of 30, 50, 70, and 100% applied at each energy level. For each patient, all monoenergetic and ASIR-V combinations were reconstructed from the same source acquisition; no additional contrast-enhanced acquisition was performed solely for reconstruction testing. The reported ASIR-V percentages represented post-acquisition reconstruction blending strengths. For example, 50% ASIR-V represented a reconstructed image containing a 50% ASIR-V contribution blended with a 50% FBP reference contribution. These post-acquisition reconstruction settings were used for noise suppression and did not directly modify tube current or radiation output during data acquisition. Higher ASIR-V blending provides stronger noise suppression but may alter image texture and reduce edge sharpness at very high levels.

For each reconstruction combination, vascular attenuation was measured in the aortic arch, common carotid artery, internal carotid artery, and M1 segment of the middle cerebral artery. Background noise, signal-to-noise ratio (SNR), and CNR were calculated using the same methods as those used in the formal study. Two radiologists with more than 10 years of CT experience independently evaluated vascular branch visibility, vessel-wall definition, enhancement uniformity, image noise, artifacts, image texture, and overall diagnostic acceptability while blinded to the reconstruction parameters. When the two radiologists differed in their assessment of a reconstruction combination, they jointly re-reviewed the corresponding image set and discussed the discrepancy with reference to the predefined qualitative evaluation criteria until a mutually agreed final assessment was reached.

Candidate combinations were evaluated using vascular attenuation, background noise, SNR, CNR, and consensus-based qualitative assessments of vessel-edge definition, branch visibility, enhancement uniformity, image noise, artifacts, image texture, and overall diagnostic acceptability. For each contrast protocol, the final reconstruction setting was selected by consensus on the basis of the combined objective and qualitative findings. The pilot findings are reported separately in Section 2.1 and summarized in Supplementary Table S1. The selected settings were regarded as protocol-specific compromises rather than universally optimal reconstruction parameters.

During the pilot phase, the effect of ASIR-V blending strength was assessed across the spectral reconstruction combinations, focusing on image noise, image texture, and vessel-edge sharpness. The corresponding findings are summarized in Supplementary Table S1.

#### 2.2.2 Formal study: spectral CTA acquisition and reconstruction protocols

The following acquisition and reconstruction protocols were applied to the spectral low-dose and spectral ultra-low-dose groups in the formal 150-participant study. Both spectral groups were acquired using the same rapid 80/140-kVp switching GSI mode with identical preset acquisition settings (11), including automatic tube-current modulation with a noise index of 10, detector configuration of 256 × 0.625 mm, a pitch of 1.375:1, a gantry rotation time of 0.5 s/rev, a field of view of 25 × 25 cm, and a slice thickness of 0.625 mm. Because automatic tube-current modulation with a fixed noise index of 10 was used, the actual tube current and radiation output were determined during acquisition according to individual patient attenuation and the acquisition mode. The two spectral protocols differed only in the selected virtual monoenergetic level, ASIR-V blending strength, and contrast administration protocol.

For the spectral low-dose group, 65-keV virtual monoenergetic images were reconstructed using 50% ASIR-V blending, and the prescribed weight-based contrast dose was 0.55 mL/kg at an injection rate of 4.5 mL/s. For the spectral ultra-low-dose group, 60-keV virtual monoenergetic images were reconstructed using 70% ASIR-V blending, and the prescribed weight-based contrast dose was 0.50 mL/kg at an injection rate of 4.0 mL/s.

Virtual non-contrast images and contrast-enhanced virtual monoenergetic images were reconstructed from the same GSI acquisition. Layer-by-layer subtraction was subsequently performed using Add/Sub software to generate post-subtraction images. Because both image sets originated from the same acquisition, this workflow minimized interscan misregistration associated with separately acquired unenhanced and enhanced series.

#### 2.2.3 Image visualization and quantitative analysis

**Figure 1:**
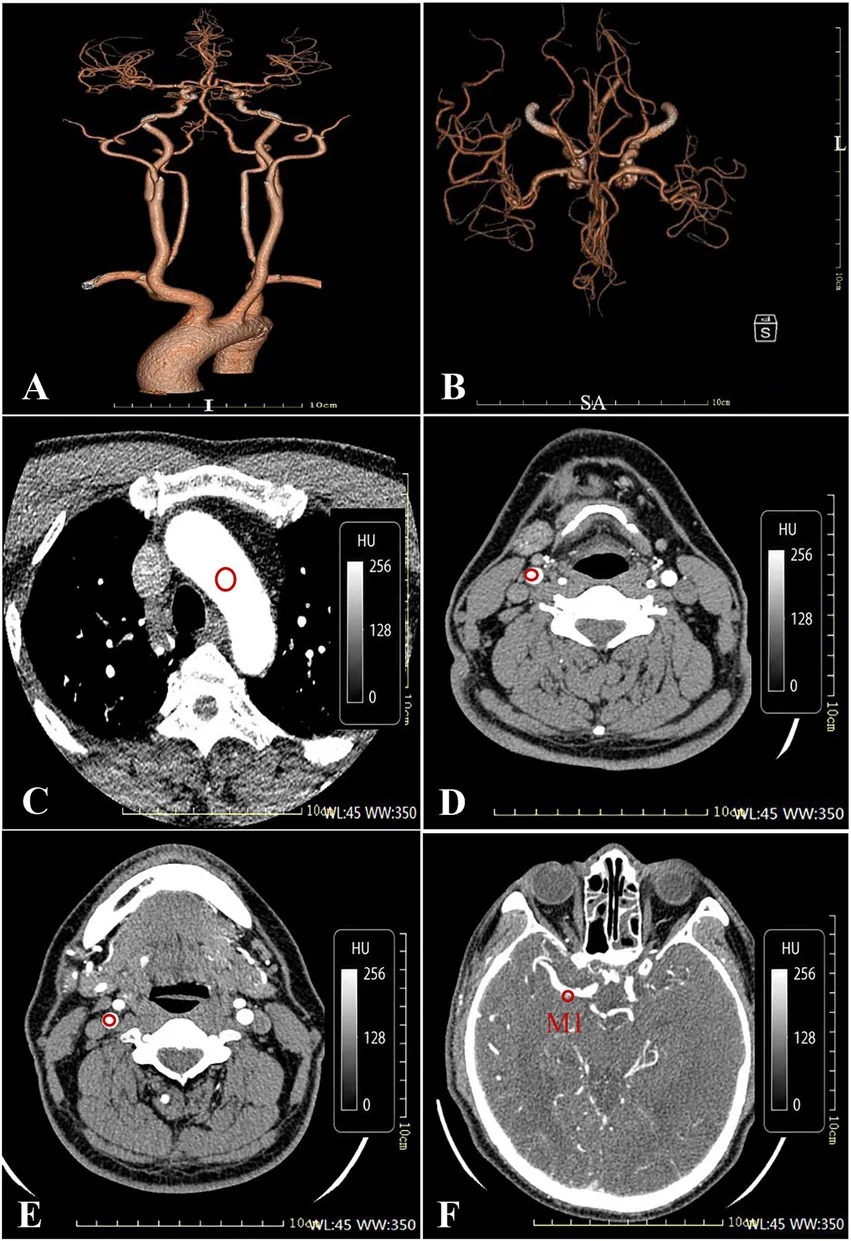
Representative head and neck CTA images from a participant in the conventional-dose group. The examination was performed using conventional 100-kVp acquisition with FBP reconstruction, a prescribed weight-based contrast dose of 0.90 mL/kg, and an injection rate of 5.0 mL/s. **(A)** Three-dimensional VR image showing the overall arterial tree from the aortic arch to the intracranial vessels. **(B)** Enlarged VR view of the intracranial arteries and circle of Willis. Matched axial source images show **(C)** the aortic arch, **(D)** the upper common carotid artery, **(E)** the lower internal carotid artery, and **(F)** the M1 segment of the middle cerebral artery. Circles indicate the vascular ROIs used for CT attenuation measurements. On this representative conventional-dose examination, the arterial tree is continuously visualized from the aortic arch to the intracranial vessels, with clear depiction of distal branches, sharp vessel margins, homogeneous enhancement, and no major artifacts. Scale bars are shown in all panels, and grayscale/HU bars are shown in the grayscale source-image panels. CTA, computed tomography angiography; VR, volume rendering; FBP, filtered back projection; ROI, region of interest; HU, Hounsfield unit.

**Figure 2:**
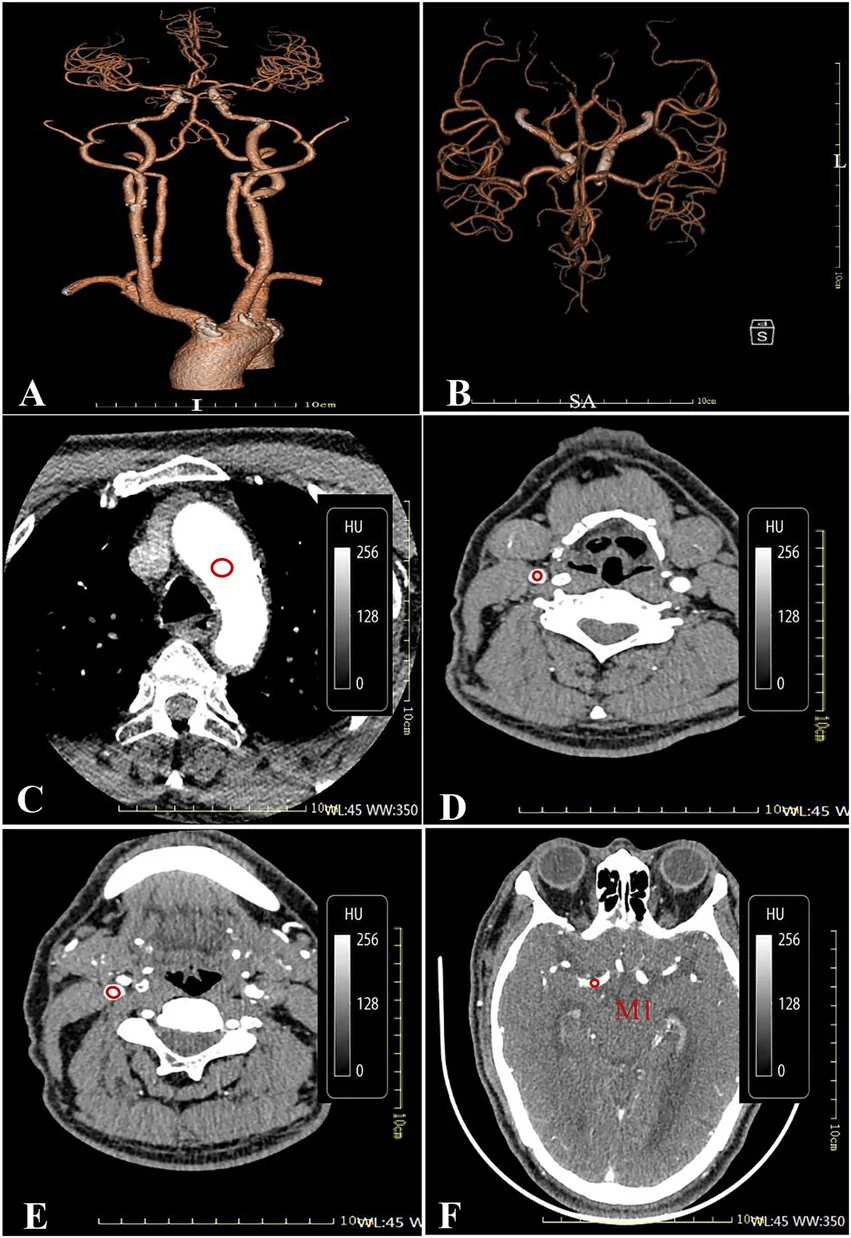
Representative head and neck CTA images from a participant in the spectral low-dose group. The examination was performed using rapid 80/140-kVp switching spectral acquisition, 65-keV virtual monoenergetic reconstruction with 50% ASIR-V blending, a prescribed weight-based contrast dose of 0.55 mL/kg, and an injection rate of 4.5 mL/s. **(A)** Three-dimensional VR image showing the overall arterial tree from the aortic arch to the intracranial vessels. **(B)** Enlarged VR view of the intracranial arteries and circle of Willis. Matched axial source images show **(C)** the aortic arch, **(D)** the upper common carotid artery, **(E)** the lower internal carotid artery, and **(F)** the M1 segment of the middle cerebral artery. Circles indicate the vascular ROIs used for CT attenuation measurements. On this representative spectral low-dose examination, vascular continuity and distal branch depiction are preserved, vessel margins remain well defined, enhancement is diagnostically adequate, and no major artifacts are evident. Scale bars are shown in all panels, and grayscale/HU bars are shown in the grayscale source-image panels. CTA, computed tomography angiography; VR, volume rendering; ASIR-V, adaptive statistical iterative reconstruction-V; ROI, region of interest; HU, Hounsfield unit.

**Figure 3:**
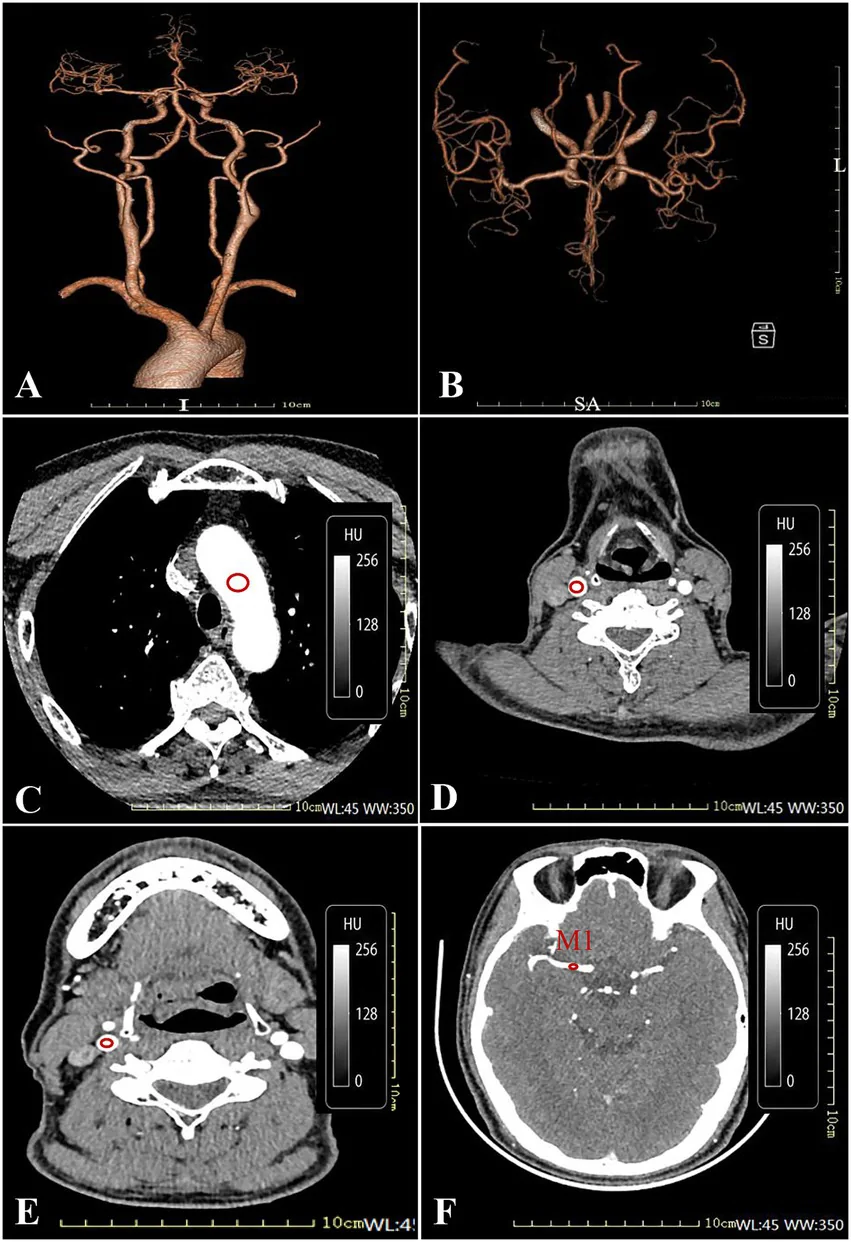
Representative head and neck CTA images from a participant in the spectral ultra-low-dose group. The examination was performed using rapid 80/140-kVp switching spectral acquisition, 60-keV virtual monoenergetic reconstruction with 70% ASIR-V blending, a prescribed weight-based contrast dose of 0.50 mL/kg, and an injection rate of 4.0 mL/s. **(A)** Three-dimensional VR image showing the overall arterial tree from the aortic arch to the intracranial vessels. **(B)** Enlarged VR view of the intracranial arteries and circle of Willis. Matched axial source images show **(C)** the aortic arch, **(D)** the upper common carotid artery, **(E)** the lower internal carotid artery, and **(F)** the M1 segment of the middle cerebral artery. Circles indicate the vascular ROIs used for CT attenuation measurements. On this representative spectral ultra-low-dose examination, vascular continuity, distal branch depiction, and vessel-edge definition remain diagnostically acceptable, with no major artifacts despite the lowest prescribed weight-based contrast dose and injection rate among the three protocols. Scale bars are shown in all panels, and grayscale/HU bars are shown in the grayscale source-image panels. CTA, computed tomography angiography; VR, volume rendering; ASIR-V, adaptive statistical iterative reconstruction-V; ROI, region of interest; HU, Hounsfield unit.

All image datasets were transferred to a GE AW 4.7 workstation. Maximum intensity projection (MIP) images were used to survey vascular continuity and branching, and multiplanar reconstruction (MPR) images were used to confirm vessel course and localize the predefined anatomical levels for axial region-of-interest (ROI) placement. Volume-rendering (VR) images were generated only for three-dimensional display and representative illustrations. For Figures 1–3, one representative examination from each group was re-exported and matched predefined anatomical levels, including the overall head and neck arterial tree, intracranial arteries, aortic arch, upper common carotid artery, lower internal carotid artery, and M1 segment of the middle cerebral artery. Image orientation, field of view, magnification, and display settings were standardized across groups. Objective quantitative image analysis was performed exclusively on axial source images by one radiologist with more than 10 years of CT experience who was blinded to the scanning protocol. Conventional polychromatic 100-kVp FBP images were used in the conventional-dose group, whereas selected 65- and 60-keV virtual monoenergetic images were used in the spectral low-dose and spectral ultra-low-dose groups, respectively.

A circular region of interest (ROI) was manually placed at the center of the vascular lumen at each predefined anatomical site, including the aortic arch, upper common carotid artery, lower internal carotid artery, and M1 segment of the middle cerebral artery. Each vascular ROI was adjusted to encompass a homogeneous portion of the contrast-enhanced blood pool and was made as large as practicable while avoiding the vessel wall, plaques, calcification, branching points, image artifacts, and partial-volume effects. ROI placement and size were kept as consistent as possible for the same anatomical site across participants and study groups. The mean attenuation value, expressed in Hounsfield units, within each vascular ROI was recorded as the vascular CT attenuation value.

For each participant, a separate 2-mm^2^ ROI was placed in a homogeneous region of the sternocleidomastoid muscle on a standardized cervical axial image corresponding to the carotid arterial measurement level. The muscle ROI was positioned to avoid visible vessels, fat, fascia, calcification, and image artifacts. The mean attenuation within the sternocleidomastoid muscle ROI was recorded as the reference muscle attenuation, and the standard deviation within the same ROI was defined as background noise (BN). The reference muscle attenuation and BN were used in the calculation of vascular SNR and CNR.

Vascular and muscle ROI measurements were repeated three times, and the mean values were used for statistical analysis. CNR was calculated as (mean vascular ROI attenuation − mean sternocleidomastoid muscle ROI attenuation)/BN, and SNR was calculated as mean vascular ROI attenuation/BN. When unilateral vascular occlusion prevented measurement, the corresponding contralateral vessel was measured.

### 2.3 Radiation and contrast agent dosage

The dose-length product (DLP) was obtained from the system-generated CT dose report. The effective dose (ED) was calculated using the formula ED = k × DLP, where k was the conversion coefficient for head and neck CT examinations (0.0023 mSv·mGy^−1^·cm^−1^). ED was expressed in millisieverts (mSv), and DLP in mGy·cm. The actual total contrast agent volume was calculated as the group-specific prescribed weight-based contrast dose (mL/kg) multiplied by the participant’s measured pre-scan body weight (kg). All participants received iopromide containing 370 mg I/mL, equivalent to 0.370 g I/mL. Accordingly, total iodine load was calculated as follows: total iodine load (g I) = actual total contrast agent volume (mL) × 0.370 g I/mL. Total iodine load was derived from actual contrast agent volume because all three groups received the same iodine concentration.

### 2.4 Image quality assessment

Subjective image quality was independently evaluated by two radiologists, each with more than 10 years of CT experience, who were blinded to the scanning protocol. Axial images were used as the primary datasets for subjective scoring, while MIP and MPR images were available only as adjuncts for evaluating vascular continuity and branch visibility. 5 points: The main trunk and first- to fourth-order branches of the target vessel were clearly visualized with smooth vessel walls, uniform enhancement, homogeneous density, and no artifacts. 4 points: The main trunk and first- to third-order branches were clearly displayed with well-defined vessel walls, adequate contrast enhancement, and relatively uniform density. 3 points: The main trunk and first- to second-order branches were clearly visualized, vessel walls were mostly smooth, contrast enhancement was acceptable, and density was fairly uniform, allowing for diagnostic interpretation. 2 points: Only the main trunk and first-order (or a few second-order) branches were visible, with irregular vessel walls, discontinuous contrast filling, heterogeneous density, and significant artifacts that affected diagnosis. 1 point: Only the main trunk and first-order branches were visible with blurred vessel walls, poor contrast enhancement, and severe artifacts, rendering the image non-diagnostic.

### 2.5 Statistical analysis

Statistical analysis was performed using SPSS version 25.0 (IBM Corp., Armonk, NY, USA). Pilot parameter-selection findings were summarized descriptively because the pilot was intended for protocol selection and because multiple reconstruction combinations were generated from the same source acquisition within each participant. The Kolmogorov–Smirnov test was used to assess the normality of continuous variables. Data following a normal distribution were expressed as mean ± standard deviation (SD) and compared among groups using one-way analysis of variance (ANOVA), with the corresponding *F* statistics and *p* values reported. Pairwise comparisons were conducted using the LSD-t test. Interobserver agreement for subjective image-quality scores between the two radiologists was evaluated using Cohen’s kappa statistic. According to the criteria proposed by Landis and Koch, *κ* < 0 indicated poor agreement; κ = 0.00–0.20, slight agreement; κ = 0.21–0.40, fair agreement; κ = 0.41–0.60, moderate agreement; κ = 0.61–0.80, substantial agreement; and κ = 0.81–1.00, almost perfect agreement (12). Categorical data were expressed as counts and percentages (%) and analyzed using the chi-square (χ^2^) test. Ordinal data were compared using the Kruskal–Wallis H test, and pairwise comparisons were conducted with the Mann–Whitney U test. A *p* value < 0.05 was considered statistically significant.

## 3 Results

### 3.1 Pilot parameter-optimization findings

In the 20-patient pilot cohort, decreasing the virtual monoenergetic level from 70 to 50–55 keV increased vascular attenuation but also increased background noise and produced a grainier image appearance, particularly in the lower neck and skull-base regions. Increasing the ASIR-V blending strength improved noise suppression; however, 100% ASIR-V produced an over-smoothed appearance and reduced vessel-edge sharpness. For the 0.55-mL/kg contrast protocol administered at 4.5 mL/s, 65 keV with 50% ASIR-V provided a favorable balance among vascular attenuation, image noise, CNR, and preservation of natural image texture. For the 0.50-mL/kg contrast protocol administered at 4.0 mL/s, 60 keV with 70% ASIR-V was selected to increase iodine conspicuity while maintaining sufficient noise suppression and vessel-edge definition. These findings are summarized in Supplementary Table S1.

### 3.2 Baseline demographic characteristics

No significant difference in sex distribution was observed among the conventional-dose, spectral low-dose, and spectral ultra-low-dose groups (32/18, 37/13, and 34/16 males/females, respectively; χ^2^ = 1.177, *p* = 0.555). One-way ANOVA also showed no significant differences in age, height, weight, or BMI among the three groups (all *p* > 0.05).

### 3.3 Comparison of objective image parameters

The CT attenuation values of the aortic arch, common carotid artery, internal carotid artery, and M1 segment of the middle cerebral artery were significantly lower in the spectral low-dose and ultra-low-dose groups than in the conventional-dose group, indicating lower quantitative vascular enhancement in the two reduced-contrast protocols. However, there were no significant differences between the spectral low-dose and ultra-low-dose groups (*p* > 0.05). The signal-to-noise ratio (SNR) and contrast-to-noise ratio (CNR) of cervical and cerebral vessels were also significantly lower in the spectral low-dose and ultra-low-dose groups than in the conventional-dose group (*p* < 0.001). No statistically significant differences were observed between the spectral low-dose and ultra-low-dose groups (*p* > 0.05). Detailed quantitative comparisons are presented in Tables 1, 2.

**Table 1:** Comparison of baseline demographic characteristics among the three groups.

Parameter	Conventional-dose group (*n* = 50)	Spectral low-dose group (*n* = 50)	Spectral ultra-low-dose group (*n* = 50)	Test statistic	*p* value
Sex, male/female, *n*	32/18	37/13	34/16	χ^2^ = 1.177	0.555
Age, years	60.60 ± 11.88	59.20 ± 11.31	56.86 ± 11.84	*F* = 1.307	0.274
Height, cm	165.66 ± 8.46	167.70 ± 6.72	168.28 ± 7.54	*F* = 1.634	0.199
Weight, kg	68.32 ± 8.96	67.22 ± 6.15	67.94 ± 6.80	*F* = 0.284	0.753
BMI, kg/m^2^	24.90 ± 2.82	23.93 ± 2.23	24.05 ± 2.56	*F* = 2.133	0.122

Sex is presented as male/female counts and was compared among the three groups using the Pearson chi-square test. Continuous variables are presented as mean ± standard deviation and were compared using one-way analysis of variance. χ^2^ denotes the Pearson chi-square statistic, and *F* denotes the one-way ANOVA statistic. No statistically significant differences in baseline demographic characteristics were observed among the three groups.

**Table 2:** Comparison of vascular CT attenuation, SNR, and CNR among the three groups.

Parameter	Conventional-dose group (*n* = 50)	Spectral low-dose group (*n* = 50)	Spectral ultra-low-dose group (*n* = 50)	*F* statistic	*p* value
Aortic Arch (HU)	563.67 ± 52.66ᵃ	440.23 ± 58.40ᵇ	430.32 ± 52.76ᵇ	92.32	< 0.001
Common Carotid Artery (HU)	589.63 ± 64.76ᵃ	445.19 ± 68.95ᵇ	440.45 ± 60.16ᵇ	85.80	< 0.001
Internal Carotid Artery (HU)	577.53 ± 65.43ᵃ	430.54 ± 69.62ᵇ	435.30 ± 60.91ᵇ	81.49	< 0.001
Middle Cerebral Artery (M1) (HU)	503.01 ± 51.96ᵃ	375.92 ± 60.62ᵇ	378.34 ± 52.36ᵇ	86.93	< 0.001
Neck Vessel SNR	63.33 ± 5.65ᵃ	48.12 ± 6.12ᵇ	49.05 ± 4.94ᵇ	116.04	< 0.001
Neck Vessel CNR	58.18 ± 6.64ᵃ	40.84 ± 6.04ᵇ	41.70 ± 4.79ᵇ	138.11	< 0.001
Brain Vessel SNR	55.52 ± 4.32ᵃ	40.71 ± 5.76ᵇ	42.14 ± 4.46ᵇ	139.22	< 0.001
Brain Vessel CNR	51.96 ± 4.04ᵃ	33.43 ± 5.52ᵇ	34.79 ± 4.31ᵇ	203.89	< 0.001

Data are expressed as mean ± standard deviation (SD). Values sharing the same superscript letter did not differ significantly, whereas values with different superscript letters differed significantly (*p* < 0.05). HU, Hounsfield unit; SNR, signal-to-noise ratio; CNR, contrast-to-noise ratio. *F* denotes the test statistic obtained from one-way analysis of variance, and *p* denotes the corresponding *p* value.

### 3.4 Comparison of subjective image quality

All examinations received subjective image-quality scores of 3–5 and were therefore considered diagnostically acceptable. Interobserver agreement for subjective image-quality scoring between the two radiologists was substantial (*κ* = 0.761, *p* < 0.05), according to the Landis and Koch criteria. A significant difference was observed in image quality scores among the three groups (*p* < 0.05). Both the spectral low-dose and spectral ultra-low-dose groups received higher subjective image-quality scores than the conventional-dose group despite their lower vascular attenuation, SNR, and CNR. These subjective scores reflect reader-assessed branch visibility, vessel-wall definition, enhancement uniformity, artifacts, and overall diagnostic acceptability. No statistically significant differences in vascular attenuation, SNR, CNR, or subjective image-quality scores were observed between the two spectral groups (*p* > 0.05), as shown in Table 3. Figures 1–3 present representative and anatomically matched head and neck CTA images from the conventional-dose, spectral low-dose, and spectral ultra-low-dose groups. Figures 1A, 2A, and 3A show the overall head and neck arterial tree; Figures 1B, 2B, and 3B show the intracranial arteries and circle of Willis; and Figures 1C–F, 2C–F, and 3C–F show matched axial source images of the aortic arch, upper common carotid artery, lower internal carotid artery, and M1 segment of the middle cerebral artery, respectively. Figures 1–3 visually complement the group-level results presented in Tables 2–4. The conventional-dose group showed higher vascular attenuation, SNR, and CNR, whereas both spectral protocols preserved continuous arterial visualization, distal branch depiction, vessel-edge definition, and overall diagnostic acceptability despite lower radiation and contrast exposure. No statistically significant differences in objective or subjective image-quality measures were observed between the two spectral protocols. The circles shown in Figures 1–3 indicate representative vascular ROI locations used for CT attenuation measurements.

**Table 3:** Comparison of subjective image-quality scores among the three groups.

Parameter	Conventional-dose group (*n* = 50)	Spectral low-dose group (*n* = 50)	Spectral ultra-low-dose group (*n* = 50)	Z	*p* value
Score 3	9 (18.0%)	2 (4.0%)	3 (6.0%)	−2.437	0.015
Score 4	26 (52.0%)	26 (52.0%)	25 (50.0%)		
Score 5	15 (30.0%)	22 (44.0%)	22 (44.0%)		

Subjective image-quality scores were independently assigned by two radiologists using a five-point grading scale. The assessment incorporated vascular branch visibility, vessel-wall definition, enhancement uniformity, artifacts, and overall diagnostic acceptability. These scores summarize reader-assessed visual interpretability.

**Table 4:** Comparison of radiation dose and contrast agent parameters among the three groups.

Parameter	Conventional-dose Group (*n* = 50)	Spectral Low-dose Group (*n* = 50)	Spectral Ultra-low-dose Group (*n* = 50)	*F* statistic	*p* value
DLP (mGy·cm)	623.36 ± 104.93	329.43 ± 54.74ᵃ	321.78 ± 50.50ᵃ	267.84	< 0.001
ED (mSv)	1.42 ± 0.24	0.75 ± 0.12ᵃ	0.74 ± 0.11ᵃ	265.81	< 0.001
Actual total contrast agent volume (mL)	61.32 ± 7.98	37.48 ± 3.42ᵃ	34.32 ± 3.40ᵃᵇ	375.56	< 0.001
Total iodine load (g I)	22.69 ± 2.95	13.87 ± 1.27ᵃ	12.70 ± 1.26ᵃᵇ	375.56	< 0.001

Values are presented as mean ± standard deviation (SD). ᵃ*p* < 0.05 compared with the conventional-dose group; ᵇ*p* < 0.05 compared with the spectral low-dose group. DLP, dose–length product; ED, effective dose. The prescribed weight-based contrast doses were 0.90, 0.55, and 0.50 mL/kg for the conventional-dose, spectral low-dose, and spectral ultra-low-dose groups, respectively. Actual total contrast agent volume was calculated as the group-specific prescribed weight-based contrast dose (mL/kg) multiplied by the participant’s measured pre-scan body weight (kg). Total iodine load was calculated as actual total contrast agent volume (mL) multiplied by the iodine concentration of iopromide (0.370 g I/mL). Because the same iodine concentration was used in all groups, total iodine load represents a direct derived measure of actual total contrast agent volume rather than an independent exposure variable. *F* denotes the test statistic obtained from one-way analysis of variance (ANOVA), and *p* denotes the corresponding *p* value.

### 3.5 Comparison of radiation dose and contrast agent usage

The dose-length product (DLP), effective dose (ED), actual total contrast agent volume, and corresponding total iodine load were significantly lower in both the spectral low-dose and spectral ultra-low-dose groups than in the conventional-dose group (*p* < 0.05). Compared with the spectral low-dose group, the spectral ultra-low-dose group showed a modest but statistically significant further reduction in actual total contrast agent volume and the corresponding derived total iodine load. However, the absolute between-group differences were relatively small and should be interpreted cautiously. No significant differences in DLP or ED were observed between the two spectral groups (*p* > 0.05). Detailed results are presented in Table 4.

## 4 Discussion

Head and neck CTA offers rapid, accurate, and multidimensional visualization of vascular structures with high spatial resolution, making it highly effective and practical for diagnosing CVD. However, conventional CTA often requires the intravenous administration of large volumes of high-concentration iodinated contrast agents to enhance vascular visibility and improve diagnostic accuracy. Excessive contrast volume or high injection rates can increase nephrotoxicity, leading to tubular epithelial cell apoptosis and necrosis, and raising the risk of contrast-induced nephropathy (CIN) (13, 14). For this reason, patients with acute or chronic nephritis or renal insufficiency are typically excluded from CTA examinations. Moreover, the use of large contrast volumes can sometimes result in subclavian venous contamination, which significantly degrades carotid image quality. Therefore, an important goal of CTA protocol optimization is to reduce contrast agent volume and lower the contrast injection rate while maintaining diagnostically acceptable image quality.

GSI employs rapid switching between low and high tube voltages during a single acquisition. This rapid switching reduces the temporal separation between the low- and high-energy projection datasets, thereby improving their temporal correspondence and reducing inter-energy temporal misregistration during material decomposition. However, rapid kVp switching does not prevent patient motion or correct motion artifacts caused by swallowing or head movement during the acquisition. In spectral CT, rapid switching between high and low tube voltages enables the acquisition of dual-energy projection data, from which 101 discrete virtual monoenergetic image sets can subsequently be reconstructed at 1-keV increments from 40 to 140 keV. Each virtual monoenergetic energy level provides distinct image characteristics. At lower virtual monoenergetic energies, iodine attenuation increases more markedly than the attenuation of surrounding soft tissues, thereby increasing iodine-related vascular contrast and vessel conspicuity. However, statistical fluctuations propagated through spectral material decomposition and image reconstruction may also be amplified at lower keV, resulting in increased image noise (15, 16). In the present study, CNR was calculated as (mean vascular attenuation − mean muscle attenuation)/background noise. Therefore, an increase in vascular contrast may coexist with an increase in image noise, and CNR will improve only when the proportional increase in vascular-to-background attenuation difference exceeds the proportional increase in image noise. Vascular contrast and image noise are distinct image properties: vascular contrast represents the difference in mean attenuation between an iodinated vessel and the surrounding reference tissue, whereas image noise represents the standard deviation of attenuation within a homogeneous background region. Their combined effect is reflected by CNR. Accordingly, the optimal virtual monoenergetic level is not necessarily the lowest energy level, but the level that provides the most favorable protocol-specific balance between iodine-related vascular contrast and image noise. This optimization significantly enhances vascular delineation and diagnostic clarity (6). Although low-energy virtual monoenergetic images may exhibit increased noise, iterative reconstruction can suppress part of this noise and thereby improve the balance between vascular contrast and noise. In the present study, ASIR-V was used primarily for noise suppression and preservation of diagnostically relevant vascular detail; it was not assumed to increase iodine-related vascular contrast directly or to eliminate all noise associated with low-keV reconstruction.

Previous clinical and phantom studies have shown that iterative reconstruction can improve noise characteristics and help preserve image quality under reduced-dose conditions (17–19). The lower radiation dose in the spectral groups resulted from the acquisition protocols, while ASIR-V supported noise reduction and preservation of diagnostically acceptable image quality. These studies provide a technical basis for interpreting our results, but their findings should be considered in the context of differences in anatomical region, patient population, and acquisition protocol. In the present study, ASIR-V was used primarily to compensate for the increased noise associated with low-energy and reduced-dose acquisitions. Therefore, the lower DLP and ED observed in the spectral groups should be attributed to the acquisition and scan-design components of the integrated protocols rather than to post-acquisition ASIR-V reconstruction. ASIR-V contributed by suppressing reconstruction noise and helping preserve diagnostically acceptable image quality under the lower-dose acquisition conditions. Sherif et al. (20) reported that 80% ASIR-V produced better image quality than 60% ASIR-V in pediatric head CT. In the direct comparison between the two spectral protocols, the 70% ASIR-V ultra-low-dose group showed no statistically significant differences in vascular attenuation, SNR, CNR, or subjective image-quality scores relative to the 50% ASIR-V low-dose group, despite receiving a lower prescribed weight-based contrast dose. The two spectral protocols showed similar objective and subjective image-quality measures, whereas both had lower vascular attenuation, SNR, and CNR than the conventional-dose protocol. Nevertheless, quantitative comparisons across studies are limited by differences in patient populations, anatomical regions, and acquisition protocols.

The pilot findings summarized in Supplementary Table S1 illustrate two related protocol-optimization trade-offs. Lower monoenergetic levels increased iodine-related vascular attenuation but also increased background noise and graininess, whereas stronger ASIR-V blending improved noise suppression but, at 100%, produced over-smoothing and reduced vessel-edge sharpness. On this basis, 65 keV with 50% ASIR-V and 60 keV with 70% ASIR-V were selected as protocol-specific compromises for the 0.55- and 0.50-mL/kg contrast protocols, respectively. These settings should not be interpreted as universally optimal reconstruction parameters. Because monoenergetic level, ASIR-V strength, prescribed weight-based contrast dose, and injection rate were modified together in the formal study, the observed findings represent the performance of the integrated imaging protocols rather than the independent effect of any single component.

Vascular CT attenuation measured in Hounsfield units reflects the degree of intraluminal contrast enhancement and is an important objective indicator of vessel conspicuity. In the present study, the attenuation values of the aortic arch, common carotid artery, internal carotid artery, and M1 segment, together with the vascular SNR and CNR values, were significantly lower in the spectral low-dose and ultra-low-dose groups than in the conventional-dose group. These findings indicate a measurable reduction in quantitative vascular enhancement, which was likely related to the lower prescribed weight-based contrast doses used in the spectral protocols.

The apparent discrepancy between the objective and subjective findings can be explained by the different image attributes evaluated. In this study, SNR and CNR were strongly influenced by vascular attenuation and therefore by the amount of intravascular iodine. The conventional-dose group received 0.9 mL/kg of contrast material, whereas the spectral low-dose and ultra-low-dose groups received 0.55 and 0.50 mL/kg, respectively. Although 60–65 keV monoenergetic reconstruction increased iodine conspicuity, it did not fully compensate for the lower prescribed weight-based contrast doses, resulting in lower vascular attenuation, SNR, and CNR. By contrast, the subjective scoring system evaluated vascular branch visibility, vessel-wall definition, enhancement uniformity, and artifacts. The combined spectral reconstruction and ASIR-V protocol likely helped preserve vessel delineation and control the visual appearance of noise and artifacts, allowing all examinations to remain diagnostically acceptable and resulting in higher reader scores. Thus, the lower objective parameters reflect reduced quantitative vascular enhancement, whereas the higher subjective scores reflect overall visual interpretability. These findings should not be interpreted as evidence of superior quantitative image quality or improved lesion-detection accuracy. These results demonstrate the trade-off between reducing contrast exposure and preserving diagnostically adequate vascular visualization.

Other contemporary CTA reconstruction approaches include full model-based iterative reconstruction and deep-learning reconstruction, which may provide different balances among image noise, vessel-edge sharpness, image texture, and reconstruction efficiency. However, their performance is scanner-, algorithm-, and task-dependent. Because these approaches were not available in the reconstruction environment used in the present study, no direct head-to-head comparison was performed.

Zhou et al. (21) demonstrated that using low-iodine contrast agents in combination with spectral CT at an optimal monochromatic energy level of 55 keV significantly improves head and neck vascular image quality. Even with reduced contrast volume, both subjective and objective image quality were maintained, confirming strong clinical applicability. Ren et al. (22) reported no significant differences in CT attenuation, SNR, or CNR between 50-keV ASIR-V images and conventional 120-kVp CTA images while achieving reductions in contrast medium and radiation dose. Our findings similarly support the feasibility of combining virtual monoenergetic imaging with ASIR-V for reduced-dose CTA; however, unlike their results, vascular attenuation, SNR, and CNR were lower in our spectral groups than in the conventional-dose group. This difference may be related to the lower weight-based contrast doses used in our study, the selected 60–65-keV reconstructions, anatomical coverage, scanner configuration, and ASIR-V blending strength. Importantly, all images remained diagnostically acceptable, indicating that the reduction in objective image parameters did not compromise clinical interpretability under the evaluated protocols. Huang et al. (23) reported that 50 keV monochromatic imaging could reduce the contrast agent by half in coronary CTA while maintaining excellent image quality. Majeed NF et al. (24) evaluated the use of monochromatic imaging in preoperative CT angiography for potential living-donor liver transplantation (LDALT) donors and identified 40 keV as the optimal energy level for maximizing vascular SNR and CNR. Their findings suggest that low-keV virtual monochromatic imaging (VMI) can improve the detection of vascular anatomical variations relevant to surgical planning. In the present study, both spectral protocols substantially reduced actual total contrast agent volume and the corresponding total iodine load compared with the conventional-dose protocol. Compared with the spectral low-dose group, the spectral ultra-low-dose group achieved a modest but statistically significant further reduction in actual total contrast agent volume and the corresponding derived total iodine load, without statistically significant differences in vascular attenuation, SNR, CNR, or subjective image-quality scores. The spectral ultra-low-dose protocol achieved a modest additional reduction in contrast volume and iodine load relative to the spectral low-dose protocol, with similar image-quality measures between the two spectral groups. The clinical relevance of this small absolute reduction requires further evaluation; the finding indicates only that the 60-keV/70% ASIR-V protocol permitted a small additional reduction in contrast administration without a detectable difference in the evaluated image-quality measures.

Si-Mohamed et al. (25) compared virtual non-contrast (VNC) imaging with conventional unenhanced CT for evaluating intramural hematoma in acute aortic syndrome. Both methods achieved high diagnostic accuracy, while the VNC technique reduced radiation exposure by approximately 40%. Jing M et al. (26) investigated the feasibility of using abdominal VNC imaging with spectral CT to evaluate chemotherapy-related fatty liver disease while reducing radiation exposure. Their results showed that the mean hepatic and splenic CT attenuation values on VNC images were significantly lower than those on true non-contrast (TNC) images (*p* < 0.05), with good consistency in the liver-to-spleen ratio (*p* > 0.05). They concluded that VNC imaging could serve as a reliable alternative to TNC imaging for CRFLD assessment, with a radiation dose reduction of up to 25%. Spectral CT achieves dual-energy acquisition through rapid single-source switching between 80 and 140 kVp (27). Using water–iodine material decomposition, it can generate iodine density maps under contrast-enhanced conditions, effectively isolating iodine from background tissues. The resulting iodine-free water density maps can serve as virtual non-contrast images (28). Previous studies have suggested that, in selected head and neck CT applications, VNC imaging may reduce the need for a separate non-contrast acquisition and may provide image quality comparable to TNC imaging (29). However, a direct diagnostic comparison between VNC and TNC images was not performed in the present study. In the present study, both spectral groups showed significantly lower DLP and ED values than the conventional-dose group. Although vascular attenuation, SNR, and CNR were lower in the spectral groups, all examinations remained diagnostically acceptable, and the spectral groups received higher reader-assessed subjective image-quality scores. These findings support the feasibility of incorporating VNC into the evaluated integrated dose-reduction workflow; however, because VNC and TNC images were not directly compared, the present study does not establish their diagnostic equivalence or interchangeability.

Head and neck CT angiography (CTA) typically requires high concentration and rapid injection of iodinated contrast agents, which increases the risk of contrast extravasation during administration. Because the contrast medium is delivered under high pressure, greater injection resistance and velocity elevate intravascular wall stress and vascular permeability, predisposing to leakage. Iodinated contrast agents are strongly hydrophilic and hyperosmolar; once extravasated into subcutaneous or fascial spaces, they can cause persistent osmotic fluid shifts, leading to localized pain, swelling, and related complications (30). Therefore, reducing the injection rate of iodinated contrast agents can markedly lower the incidence of extravasation and improve patient safety. Feng R et al. (31) employed a coronary CTA protocol using 70 kVp combined with a reduced injection rate (3.5 mL/s) and lower contrast volume (28 mL). Their results demonstrated that image quality and diagnostic accuracy were comparable to those obtained with a conventional 5 mL/s injection rate and 40 mL of contrast, showing that dose reduction does not compromise diagnostic performance. The spectral low-dose and spectral ultra-low-dose protocols used lower prescribed contrast injection rates of 4.5 and 4.0 mL/s, respectively, than the conventional-dose protocol of 5.0 mL/s. No significant difference in subjective image-quality scores was observed between the two spectral groups, and all examinations remained diagnostically acceptable. These findings suggest that the selected virtual monoenergetic reconstructions combined with ASIR-V permitted lower prescribed contrast doses and injection rates while preserving diagnostic acceptability.

Compared with previous studies, the present study provides a systematic evaluation of an integrated protocol combining selected 60–65-keV virtual monoenergetic imaging, 50–70% ASIR-V blending, and virtual non-contrast imaging. It also provides preliminary evidence for the feasibility of a 0.50-mL/kg contrast protocol in head and neck CTA on the evaluated CT platform. Because patients with renal insufficiency were excluded and renal safety outcomes were not assessed, the applicability of this protocol to patients with impaired renal function requires dedicated prospective validation.

Limitations: Several limitations of this study should be acknowledged. First, this was a single-center study with a relatively limited sample size, and all examinations were performed using a single CT platform. Second, the parameter-optimization pilot included only 20 participants and was designed as a descriptive protocol-selection exercise rather than a confirmatory comparison. Although the principal objective trends and consensus qualitative findings are now summarized in Supplementary Table S1, the limited sample size and protocol-specific design do not establish universally optimal reconstruction settings. Only the two selected monoenergetic levels and ASIR-V blending strengths were subsequently evaluated prospectively in the formal study. Third, monoenergetic level, ASIR-V strength, prescribed weight-based contrast dose, and injection rate were varied simultaneously; therefore, the independent contribution of each component could not be determined. The cross-reconstruction comparison was descriptive, and ASIR-V was not directly compared with model-based or deep-learning reconstruction. Fourth, lesion-level diagnostic accuracy, vessel-edge sharpness, noise texture, and artifact severity were not quantitatively evaluated. Fifth, VNC and TNC images were not directly compared within the same participants, and no lesion-level diagnostic-performance analysis of VNC was performed. Therefore, the present study cannot establish diagnostic equivalence or interchangeability between VNC and TNC imaging. Dedicated prospective studies are required to determine whether VNC can serve as a substitute for TNC for specific clinical diagnostic tasks. Finally, motion artifacts were not separately quantified or compared among the groups. Future multicenter studies using wider parameter ranges and factorial or within-patient comparisons are warranted.

## 5 Conclusion

In summary, compared with the conventional CTA protocol, the evaluated integrated spectral CT protocols incorporating selected virtual monoenergetic imaging, ASIR-V reconstruction, and VNC imaging were associated with lower radiation exposure and contrast agent use while maintaining diagnostically acceptable CTA image quality. Because VNC and TNC images were not directly compared, these findings should not be interpreted as evidence of diagnostic equivalence between them. Compared with the 65-keV/50% ASIR-V protocol, the 60-keV/70% ASIR-V protocol permitted a modest further reduction in actual total contrast agent volume without statistically significant differences in vascular attenuation, SNR, CNR, or subjective image-quality scores.

## Data Availability

The original contributions presented in the study are included in the article/Supplementary material, further inquiries can be directed to the corresponding author.

## References

[1] AbuRahma AF Avgerinos ED Chang RW Darling RC 3rd Duncan AA Forbes TL . Society for Vascular Surgery clinical practice guidelines for management of extracranial cerebrovascular disease. J Vasc Surg. (2022) 75:4s–22s. doi: 10.1016/j.jvs.2021.04.073, 34153348

[2] Hijazi Z Yassi N O'Brien JT Watson R. The influence of cerebrovascular disease in dementia with Lewy bodies and Parkinson's disease dementia. Eur J Neurol. (2022) 29:1254–65. doi: 10.1111/ene.15211, 34923713

[3] Frush DP Applegate K. Computed tomography and radiation: understanding the issues. J Am Coll Radiol. (2004) 1:113–9. doi: 10.1016/j.jacr.2003.11.012, 17411538

[4] Schneider U Schäfer B. Model of accelerated carcinogenesis based on proliferative stress and inflammation for doses relevant to radiotherapy. Radiat Environ Biophys. (2012) 51:451–6. doi: 10.1007/s00411-012-0433-x, 22899337

[5] Little MP Azizova TV Hamada N. Low- and moderate-dose non-cancer effects of ionizing radiation in directly exposed individuals, especially circulatory and ocular diseases: a review of the epidemiology. Int J Radiat Biol. (2021) 97:782–803. doi: 10.1080/09553002.2021.1876955, 33471563 PMC10656152

[6] D'Angelo T Lenga L Arendt CT Bucher AM Peterke JL Caruso D . Carotid and cerebrovascular dual-energy computed tomography angiography: optimization of window settings for virtual monoenergetic imaging reconstruction. Eur J Radiol. (2020) 130:109166. doi: 10.1016/j.ejrad.2020.109166, 32693314

[7] Zhao Y Li D Liu Z Geng X Zhang T Xu Y. Comparison of image quality and radiation dose using different pre-ASiR-V and post-ASiR-V levels in coronary computed tomography angiography. J Xray Sci Technol. (2021) 29:125–34. doi: 10.3233/XST-200754 33164983

[8] Barrett T Bowden DJ Shaida N Godfrey EM Taylor A Lomas DJ . Virtual unenhanced second generation dual-source CT of the liver: is it time to discard the conventional unenhanced phase? Eur J Radiol. (2012) 81:1438–45. doi: 10.1016/j.ejrad.2011.03.042, 21501940

[9] Sauter AP Muenzel D Dangelmaier J Braren R Pfeiffer F Rummeny EJ . Dual-layer spectral computed tomography: virtual non-contrast in comparison to true non-contrast images. Eur J Radiol. (2018) 104:108–14. doi: 10.1016/j.ejrad.2018.05.007, 29857855

[10] Faul F Erdfelder E Lang AG Buchner A. G*power 3: a flexible statistical power analysis program for the social, behavioral, and biomedical sciences. Behav Res Methods. (2007) 39:175–91. doi: 10.3758/BF03193146, 17695343

[11] So A Hsieh J Imai Y Narayanan S Kramer J Procknow K . Prospectively ECG-triggered rapid kV-switching dual-energy CT for quantitative imaging of myocardial perfusion. J Am Coll Cardiol Img. (2012) 5:829–36. doi: 10.1016/j.jcmg.2011.12.026, 22897997

[12] Landis JR Koch GG. The measurement of observer agreement for categorical data. Biometrics. (1977) 33:159–74. doi: 10.2307/2529310, 843571

[13] Moriya H Mochida Y Ishioka K Oka M Maesato K Yamano M . Impact of contrast-induced nephropathy on long-term renal function after coronary angiography and contrast-enhanced computed tomography. Cardiol Cardiovasc Med. (2022) 6:473–9. doi: 10.26502/fccm.92920285, 36212510 PMC9536241

[14] Rowe AS Hawkins B Hamilton LA Ferrell A Henry J Wiseman BF . Contrast-induced nephropathy in ischemic stroke patients undergoing computed tomography angiography: CINISter study. J Stroke Cerebrovasc Dis. (2019) 28:649–54. doi: 10.1016/j.jstrokecerebrovasdis.2018.11.012, 30527789

[15] Neuhaus V Große Hokamp N Abdullayev N Maus V Kabbasch C Mpotsaris A . Comparison of virtual monoenergetic and polyenergetic images reconstructed from dual-layer detector CT angiography of the head and neck. Eur Radiol. (2018) 28:1102–10. doi: 10.1007/s00330-017-5081-8, 29018958

[16] Tao S Rajendran K Zhou W Fletcher JG McCollough CH Leng S. Improving iodine contrast to noise ratio using virtual monoenergetic imaging and prior-knowledge-aware iterative denoising (mono-PKAID). Phys Med Biol. (2019) 64:105014. doi: 10.1088/1361-6560/ab17fa, 30970337 PMC6598704

[17] Tang H Liu Z Hu Z He T Li D Yu N . Clinical value of a new generation adaptive statistical iterative reconstruction (ASIR-V) in the diagnosis of pulmonary nodule in low-dose chest CT. Br J Radiol. (2019) 92:20180909. doi: 10.1259/bjr.20180909, 31469289 PMC6849666

[18] Chen B Ramirez Giraldo JC Solomon J Samei E. Evaluating iterative reconstruction performance in computed tomography. Med Phys. (2014) 41:121913. doi: 10.1118/1.4901670, 25471973

[19] Miéville FA Gudinchet F Brunelle F Bochud FO Verdun FR. Iterative reconstruction methods in two different MDCT scanners: physical metrics and 4-alternative forced-choice detectability experiments--a phantom approach. Physica Medica. (2013) 29:99–110. doi: 10.1016/j.ejmp.2011.12.004, 22217444

[20] Sherif FM Said AM Elsayed YN Elmogy SA. Value of using adaptive statistical iterative reconstruction-V (ASIR-V) technology in pediatric head CT dose reduction. Egypt J Radiol Nucl Med. (2020) 51:164. doi: 10.1186/s43055-020-00291-2

[21] Zhou X Cui M Liu Y Wu Y Hu D Zhai D . Low dose iodinated contrast material and radiation for virtual monochromatic imaging in Craniocervical dual-layer spectral detector computed tomography angiography: a prospective and randomized study. Acad Radiol. (2024) 31:2501–10. doi: 10.1016/j.acra.2023.12.004, 38135625

[22] Ren H Zhen Y Gong Z Wang C Chang Z Zheng J. Feasibility of low-dose contrast media in run-off CT angiography on dual-layer spectral detector CT. Quant Imaging Med Surg. (2021) 11:1796–804. doi: 10.21037/qims-20-925, 33936965 PMC8047342

[23] Huang X Gao S Ma Y Lu X Jia Z Hou Y. The optimal monoenergetic spectral image level of coronary computed tomography (CT) angiography on a dual-layer spectral detector CT with half-dose contrast media. Quant Imaging Med Surg. (2020) 10:592–603. doi: 10.21037/qims.2020.02.17, 32269920 PMC7136738

[24] Majeed NF Ali SM Therrien J Wald C Wortman JR. Virtual monoenergetic spectral detector CT for preoperative CT angiography in liver donors. Curr Probl Diagn Radiol. (2022) 51:517–23. doi: 10.1067/j.cpradiol.2021.10.001, 34839975

[25] Si-Mohamed S Dupuis N Tatard-Leitman V Rotzinger D Boccalini S Dion M . Virtual versus true non-contrast dual-energy CT imaging for the diagnosis of aortic intramural hematoma. Eur Radiol. (2019) 29:6762–71. doi: 10.1007/s00330-019-06322-5, 31264015

[26] Jing M Sun J Xi H Liu Z Zhang S Deng L . Abdominal virtual non-contrast images derived from energy spectrum CT to evaluate chemotherapy-related fatty liver disease. Quant Imaging Med Surg. (2023) 13:669–81. doi: 10.21037/qims-22-742, 36819287 PMC9929393

[27] Gadsbøll EL Aurumskjöld ML Holmquist F Baubeta E. Virtual non-contrast images in photon-counting computed tomography: impact of different contrast phases. Acta Radiol. (2024) 65:1147–52. doi: 10.1177/02841851241271202, 39140849

[28] Verstraeten S Ansems J Ommen WV Linden DV Looijmans F Tesselaar E. Comparison of true non-contrast and virtual non-contrast images in the characterization of renal lesions using detector-based spectral CT. Br J Radiol. (2023) 96:20220157. doi: 10.1259/bjr.20220157, 37334964 PMC10461284

[29] Kessner R Sommer J Große Hokamp N Laukamp KR Nayate A. Virtual versus true non-contrast images of the brain from spectral detector CT: comparison of attenuation values and image quality. Acta Radiologica. (2023) 64:776–83. doi: 10.1177/02841851221093763, 35505585

[30] Sun YX Shang J Cui Y Zhang KJ Li XT Li DN . Impact of different concentration iodinated contrast media on pain and comfort in abdominal computed tomography. Eur J Radiol. (2024) 179:111664. doi: 10.1016/j.ejrad.2024.111664, 39121745

[31] Feng R Tong J Liu X Zhao Y Zhang L. High-pitch coronary CT angiography at 70 kVp adopting a protocol of low injection speed and low volume of contrast medium. Korean J Radiol. (2017) 18:763–72. doi: 10.3348/kjr.2017.18.5.763, 28860894 PMC5552460

