# Supplementary material for: Study on the combined application of selected virtual monoenergetic imaging, virtual non-contrast imaging, and adaptive statistical iterative reconstruction-V in head and neck CT angiography using 256-slice wide-body CT
Source: Front Med (Lausanne). 2026 Aug 24;13:1872871. doi: 10.3389/fmed.2026.1872871 (PMC13547066; doi:10.3389/fmed.2026.1872871)
Supplement: Supplementary file 1 [file Table_1.docx]

**Supplementary Table S1 Concise qualitative summary of the pilot reconstruction parameter-selection findings**

| **Pilot comparison or setting** | **Evaluated parameters** | **Objective trend** | **Consensus qualitative finding** | **Implication for final selection** |
| --- | --- | --- | --- | --- |
| Virtual monoenergetic energy, both spectral contrast protocols | 50, 55, 60, 65, and 70 keV at each ASIR-V strength | Vascular attenuation increased as the monoenergetic level decreased; background noise became markedly greater at 50–55 keV. | The 50–55-keV images showed a grainier appearance, particularly in the lower neck and skull-base regions. | The lowest energy levels were not selected because the gain in iodine-related vascular conspicuity was accompanied by an unfavorable increase in noise and graininess. |
| ASIR-V blending strength, both spectral contrast protocols | 30%, 50%, 70%, and 100% at each monoenergetic level | Noise suppression increased with increasing ASIR-V blending and was more apparent in low-energy spectral images. | At 100% ASIR-V, images appeared over-smoothed and vessel-edge sharpness was reduced. | Intermediate ASIR-V strengths were preferred to balance noise suppression with preservation of natural image texture and vessel-edge definition. |
| Spectral low-dose pilot protocol | 0.55 mL/kg at 4.5 mL/s; final candidate: 65 keV/50% ASIR-V | The 65-keV/50% ASIR-V combination provided a favorable balance among vascular attenuation, background noise, and CNR. | Natural image texture was preserved, and the combination was judged to provide the most favorable overall balance among the settings evaluated. | 65 keV with 50% ASIR-V was selected for the spectral low-dose protocol. |
| Spectral ultra-low-dose pilot protocol | 0.50 mL/kg at 4.0 mL/s; final candidate: 60 keV/70% ASIR-V | The lower 60-keV setting increased iodine conspicuity, while 70% ASIR-V provided sufficient noise suppression. | Sufficient noise suppression and preservation of vessel-edge definition were achieved without using 100% ASIR-V. | 60 keV with 70% ASIR-V was selected for the spectral ultra-low-dose protocol. |

The pilot cohort included 20 patients who were not included in the formal study cohort; 10 patients underwent the 0.55-mL/kg, 4.5-mL/s protocol and 10 underwent the 0.50-mL/kg, 4.0-mL/s protocol.
